# Why Treatment Fails in Type 2 Diabetes

**DOI:** 10.1371/journal.pmed.0050215

**Published:** 2008-10-28

**Authors:** Anders Rosengren, Xingjun Jing, Lena Eliasson, Erik Renström

## Abstract

Erik Renström and colleagues discuss a new mouse study that explores the mechanism behind the secondary failure of sulfonylurea treatment.

Diabetes mellitus is the most common endocrine disease in the world. The World Health Organization estimates that the disease is responsible for about 5% of all deaths globally each year, a figure that is projected to increase by 50% within a decade (http://www.who.int/diabetes/en/). Diabetes mellitus is easily diagnosed by the characteristic chronic elevation in blood glucose concentration, but is in fact merely an umbrella diagnosis with several disease subtypes.

## Failing Insulin Secretion in Type 2 Diabetes

These subtypes of diabetes mellitus are remarkably different in terms of pathogenic mechanisms and severity of disease, but converge on the insufficient release of the glucose-lowering hormone insulin in the beta-cells of the pancreatic islets. The by far most common disease variant, obesity-related type 2 diabetes, also follows this final pathogenic pathway, as clearly shown by the United Kingdom Prospective Diabetes Study [1] and recently underscored by genome-wide association scans that identified an array of pancreatic islet-related genes associating with type 2 diabetes (reviewed in [2]). Accordingly, sulfonylureas, a group of insulin secretagogues, have long been cornerstones in the pharmacological treatment of type 2 diabetes. These compounds bypass the normal glucose-sensing mechanism in the pancreatic beta-cells and thereby initiate insulin secretion. This effect is mediated by closure of the ATP-sensitive potassium channels (K_ATP_ channels) in the beta-cell membrane, leading to membrane depolarization, opening of voltage-gated Ca^2+^ channels, and finally Ca^2+^-dependent exocytosis of insulin granules [3,4]. As a result, blood glucose concentrations decrease, the risk for secondary vascular complications is lowered, and patients experience increased quality of life [5].

**Linked Research Article**

This Perspective discusses the following new study published in *PLoS Medicine:*

Remedi MS, Nichols CG (2008) Chronic antidiabetic sulfonylureas in vivo: Reversible effects on mouse pancreatic β-cells. PLoS Med 5(10): e206. doi:10.1371/journal.pmed.0050206

In a mouse study aiming to understand why long-term treatment for type 2 diabetes with sulfonylureas eventually fails, Colin Nichols and Maria Remedi suggest that slow restoration of insulin secretion may be possible after a drug-resting period.

## Secondary Failure of Sulfonylurea Treatment

Unfortunately, this story does not end on a high note. Within a few years of starting treatment with sulfonylureas, the beta-cells show clear signs of fatigue leading to deteriorated blood glucose control. Eventually all patients need to take daily insulin injections to achieve acceptable control over blood glucose [6]. The reasons underlying this *secondary failure* have long been debated. One hypothesis that has attracted considerable interest is the notion that sulfonylurea-mediated hyperexcitation of beta-cells may trigger excitotoxic reactions leading to increased rates of beta-cell apoptosis [7]. As a result, beta-cell mass decreases, and this is seen as the major cause of the developing insulin deficiency [8].

An alternative scenario is suggested by findings in mouse models. Mice with reduced K_ATP_ channel activity show the expected increased beta-cell electrical activity and, consequently, hypersecretion of insulin [9]. Mouse models with complete lack of functional K_ATP_ channels have been generated by ablation of either the K^+^ channel pore KIR6.2 or the regulatory sulfonylurea receptor 1. Surprisingly, both models exhibit a paradoxical undersecretory phenotype, in spite of continuous beta-cell electrical activity and an apparently normal pancreatic beta-cell mass [10–12]. These findings suggest that a bell-shaped relationship exists between electrical activity and insulin secretion, and that hyperexcitation leads to beta-cell failure without beta-cell death.

## Using Slow-Release Sulfonylurea Pellets in a New In Vivo Model

In this issue of *PLoS Medicine*, Remedi and Nichols have put the latter hypothesis up for scrutiny [13]. To this end, the authors used a new approach and implanted slow-release pellets with the sulfonylurea glibenclamide into normal mice. After initial stimulation of insulin release, the mice quickly developed insulinopenia and glucose intolerance, and within one week their phenotype was reminiscent of that of mice without functional K_ATP_ channels. Glucose-stimulated insulin secretion was greatly reduced in pancreatic islets freshly isolated from glibenclamide-treated mice.

The most significant finding of this study is the observation that the decreased capacity for insulin secretion is readily reversible. In isolated islets it was restored within a few hours after wash-out of the drug, and in vivo the mice regained glucose tolerance one month after cessation of glibenclamide treatment. These observations, together with the failure to detect any signs of increased beta-cell apoptosis in glibenclamide-treated mice, clearly suggest that the suppression of insulin secretion is a reversible and *functional* phenomenon, i.e., a temporary impairment of the beta-cell stimulus-secretion coupling.

## Pros and Cons of the Present Study

The approach chosen in this study is deceptively simple and may at first not appear to be strikingly innovative. However, one major advantage over genetically modified mouse models is the absence of any effects during pancreas development. For example, in K_ATP_ channel knock-out mice it is difficult to rule out the possibilities that the developing beta-cell when excessively stimulated may react by (1) increasing the capacity for Ca^2+^ buffering and/or extrusion, or (2) changing expression of any of the myriad of proteins involved in regulated exocytosis of the insulin granules. That said, this new study calls for future detailed studies to allow identification of a concrete mechanism explaining the reversible suppression of insulin secretion by long-term glibenclamide treatment.

## Clinical Implications

Another attractive feature of the experimental model used here is the similarity to the treatment given to patients with type 2 diabetes. The possible clinical implications of this study are that failing insulin secretion in type 2 diabetes should not be treated with pharmacological compounds that stimulate insulin release in a tonic fashion. Instead, preference should be given to compounds with short half-life in the circulation and compounds that enhance normal pulsatile and phasic insulin secretion. Remedi and Nichols' study should prompt further clinical studies exploring the possible advantage of such compounds for maintaining an adequate capacity for insulin secretion in type 2 diabetes. However, mice are not men, and the experimental conditions in this new study do not fully mimic the clinical situation in humans. For instance, secondary failure in mouse seems to have a much more rapid onset than in humans. Furthermore, clinical experience does not suggest that termination of sulfonylurea treatment leads to revitalization of insulin secretion in patients with secondary failure. These reservations notwithstanding, this study provides strong evidence for the view that previously neglected mechanisms are in operation during progression of type 2 diabetes, and represents an important point of embarkation for future work.
